# Microbiological Assessment of the Quality of Some Commercial Products Marketed as *Lactobacillus crispatus*-Containing Probiotic Dietary Supplements

**DOI:** 10.3390/microorganisms7110524

**Published:** 2019-11-03

**Authors:** Francesco Di Pierro, Valeria Polzonetti, Vania Patrone, Lorenzo Morelli

**Affiliations:** 1 School of Biosciences and Veterinary Medicine, University of Camerino, 62032 Camerino, Italy; valeria.polzonetti@unicam.it; 2 Scientific Dept., Velleja Research, 20100 Milan, Italy; 3 Department for Sustainable Food Process-DiSTAS, University Cattolica del Sacro Cuore, 29100 Piacenza, Italy; vania.patrone@unicatt.it (V.P.); lorenzo.morelli@unicatt.it (L.M.)

**Keywords:** probiotics, viability, molecular typing, community state types

## Abstract

In the last decade, many authors have reported low viability for probiotic products. Investigators commonly find they are not meeting claimed active counts and/or incorrect species and/or strains have been identified. We have therefore decided to verify viability, the real dose and species correspondence in nine probiotic products (seven nutritional supplements and two medical devices) collected from the Italian and French markets claiming to contain at least one strain of *L. crispatus* among the different species/strain included in the formulation. In fact, the medical relevance of *L. crispatus* strains has recently grown., as evaluating the possible dominance clusters typical of the vaginal microbiota, the Community State Type I, the one dominated by *L. crispatus*, appears to be “protective” in terms of infections, fertility and gestational duration of pregnancy. The results obtained demonstrate the generally poor quality of probiotics. Out of nine products, only two definitely contained viable *Lactobacillus crispatus* cells with a daily dose of at least 1 × 10^9^ CFU/g and with an acceptable correspondence with what is declared on the label. Among these two, only one was found to be formulated with a strain (M247) that has been scientifically documented.

## 1. Introduction

Probiotics, defined by a consensus panel of experts in 2014 as “live microorganisms that when administered in adequate amounts confer a health benefit on the host”, are considered to be endowed with properties capable of enhancing health and reducing the risk of disease [1]. The official definition does not clearly indicate the adequate daily amount content of probiotic consumption. Nonetheless, for instance, Italy and Canada regulations compel 1 × 10^9^ colony-forming units (CFU) as minimum dose [2]. Indeed, the health benefits of probiotics have been studied in a number of controlled human trials, which document a wide range of benefits [2]. Moreover, the public’s growing interest in probiotics as a tool to enhance quality of life has strongly prompted the probiotics market to expand, and analysis forecasts confirm increasing sales [3]. For all these reasons, new probiotic strains are being continuously selected and formulated in finished forms by the dairy and pharma industries. Nevertheless, many authors have often reported low quality with respect to probiotic products; from not meeting claimed active counts to incorrect species and/or strain identification [4,5,6,7,8,9]. Of course, low-quality profiles reduce both professional and consumer confidence in probiotics and instill the idea that anyone, even those with low-grade competence, can develop, manufacture, promote and sell probiotics. This situation, if not properly addressed, will likely determine in the future a decrease in probiotic use and sales. This threat is so real that it has prompted the International Scientific Association for Probiotics and Prebiotics (ISAPP) to recommend that companies undergo third-party certification for their probiotic products [10]. Among the many quality issues for probiotics, one of the most common is the maintenance of viability during packaging, storage, distribution and retailing. Survival and viability are indeed affected by a number of environmental factors such as carrier material, compression strengths, oxygen, temperature, water activity, co-presence of other ingredients and storage time, which are not always well controlled [11]. Nevertheless, even though this is all well-known, it is quite common, for instance, to find finished probiotic products on a pharmacy shelf with the storage instructions declaring they can be stored at room temperature with the stability of the strain/s at the declared temperature until the expiry date unverified. In addition, the routine uses of molecular biology to assess the real bacterial composition is still limited [12,13]. While the quality of probiotic products designed to be active in the gastrointestinal tract has been addressed, as pointed out above, by a number of papers, there is a total lack of data, as far as the authors are aware, regarding the quality of products intended to be beneficial for the urogenital tract.

In the last 10 years or so, a body of evidence has shown that the vaginal microbiota can likely be clustered into five different groups, commonly known as community state types (CSTs), listed by the use of Roman numerals from I to V, where CST I corresponds to a microbiota dominated by *Lactobacillus crispatus*, CST II by *Lactobacillus gasseri*, CST III by *Lactobacillus iners*, CST V by *Lactobacillus jensenii* and CST IV corresponds to a microbiota characterized by a very low lactobacilli content [14]. The evidence also seems to suggest a correlation between CST I and a lower risk for women of recurrent vaginosis and vulvovaginitis, papilloma virus persistence and preterm delivery [15]. This evidence has prompted the scientific community to select strains of *L. crispatus* as new probiotic candidates for vaginal well-being [16]. Therefore, in our study we have decided to analyze, to the best of our knowledge for the first time, the quality of nine probiotic products, seven nutritional supplements, and two medical devices in order to investigate whether they contain at least one strain of *L. crispatus*. This will allow us to verify if physicians and consumers can have a well-placed trust for this type of probiotic, designed to positively influence the vaginal microbiota in favor of CST I.

## 2. Materials and Methods

### 2.1. Collection of Samples

To evaluate the survival of *L. crispatus* strains, nine different marketed probiotic products, seven (A, B, C, D, F, G, and H in Table 1) nutritional supplements (NS) and two (E and I in Table 1) medical devices (MDs), were collected from pharmacies and online shops. Products were purchased at the same time, and microbiological analyses were performed three times independently from the same lot. It should be noted that the MDs were subjected to all of the analysis but they were not considered within the discussion as food supplements. Two of the nine selected products were sold in France while the others were available on the Italian market. The seven probiotic-based food supplements were both mono- and multi-strain; the MD product I was labelled three other species of lactobacilli beyond the declared strain of *L. crispatus*; the MD product E was labelled mono-strain. Details of the microbiological composition and label information for all the products are reported in Table 1. 

### 2.2. Viable Cell Number Counts

In order to assess the viable counts declared on the label, 1 g of all products was accurately weighed and resuspended in 9 mL of Maximum Recovery Diluent (MRD, Sigma-Aldrich, Milan, Italy), serially diluted and plated on different media following the manufacturer instructions, accordingly to Istituto Superiore di Sanità (ISTISAN) 2008/36: 35–63 and ISO 20128:2006. Media were selected on the basis of the *Lactobacillus* spp. composition of products. MRS agar (DeMan Rogosa Sharpe, BD, Difco, Milan, Italy) was used for the total viable count, MRS agar supplemented with 0.1 and 10 µg/mL clindamycin and ciprofloxacin was used for the enumeration of *L. crispatus* and *L. gasseri*, and MRS agar supplemented with 10 µg/mL vancomycin was used for the selection of *L. rhamnosus* and *L. reuteri* (for all antibiotics, Sigma-Aldrich, Milan, Italy) [17]. The inoculated plates were incubated at 37 °C for 72 h under anaerobic conditions. Viable counts were performed in triplicate for all considered conditions.

### 2.3. Isolation and Extraction of L. crispatus Colony DNA, 16S rRNA Gene Amplification and Molecular Typing

Colonies grown on MRS agar or MRS supplemented with clindamycin/ciprofloxacin showing the typical *L. crispatus* morphology were selected, replica-plated and lysed for DNA extraction with the Whatman™ CloneSaver™ Card System (96-well format, GE Healthcare, VWR, Milan, Italy) (VWR). DNA extraction was completed following an adapted protocol that was previously described [18]. Briefly, 5 µL of the bacterial culture were spotted on the DNA save card, 2.0 mm were punched out of each filter using a Harris Micro-Punch (Whatman Inc.) and transferred into sterile 200 µL Eppendorf tubes. Cut disks were washed three times for 10 min at room temperature with 200 µL of FTA (GE Healthcare, VWR, Milan, Italy) and washed twice for 5 min with 180 µL of TE buffer. Disks were completely dried before PCR amplification. The identification of the *L. crispatus* species was assessed by a specific PCR protocol for the 16S rRNA gene using primers and conditions previously described [19]. The LMG 9479-*L. crispatus* type strain DNA was used as the PCR positive control. The genomic DNA of the five *L. crispatus* strains isolated from plates was analyzed by RAPD (Random Amplification of Polymorphic DNA)-PCR using the RAPD2 (5′-AGC AGC GTC G-3′) primer [20]. The RAPD reaction was performed in the Applied Biosystems™ MiniAmp™ Plus Thermal Cycler (Thermo Fisher Scientific, Waltham, MA, USA); the reaction was carried out in a 25 μL final volume composed of 24 μL of Megamix (Microzone, Stourbridge, UK), 1 μL of 50 μM RAPD2 primer and the Whatman disk as template DNA. The thermal protocol consisted of an initial denaturation step (94 °C, 5 min) followed by 45 cycles of denaturation (94 °C, 30 s), annealing (55 °C, 30 s) and extension (72 °C, 30 s), and a single final extension step (72 °C, 10 min) [21]. For the visualization of DNA fingerprint patterns, 15 μL of each PCR reaction were loaded on 2.5% agarose gel.

### 2.4. Total DNA Extraction from Finished Products

The total bacterial genomic DNA was determined from 50 mg of all probiotic products using the FastDNA™ SPIN Kit for Soil (MP Biomedicals, Illkirch-Graffenstaden, France) following the manufacturer’s instructions [22]. The total DNA was amplified by the same couple of primers and the thermal protocol used for the species-specific PCR on the 16S rRNA that was performed on single colonies [19].

## 3. Results

### 3.1. Viable Cell Number Counts

All products were counted on MRS agar to determine the total viable count of lactobacilli, on MRS agar supplemented with 0.1 and 10 µg/mL clindamycin and ciprofloxacin for the selection of *L. crispatus* and *L. gasseri* species (ISO 20128:2006) and MRS agar supplemented with 10 µg/mL of vancomycin for the isolation of *L. rhamnosus* and *L. reuteri* (ISTISAN 2008/36: 35–63). The results obtained are reported in Table 2 as log-transformed mean value and the standard deviation (SD) of three independent replicates. Regarding Product A, declared to contain only a strain of *L. crispatus*, the correspondence is apparently respected obtaining 10.4 ± 0.2 Log(CFU/gr) for MRS medium, as 10.4 ± 0.4 Log(CFU/gr) for MRS supplemented with clindamycin and ciprofloxacin and no viable colonies in presence of vancomycin. The same assumption can be made for Product B, declared to contain *L. gasseri* with the recovery of 9.4 ± 0.1 Log(CFU/gr) and 8.9 ± 0.1 Log(CFU/gr), respectively for MRS medium and for its supplementation with clindamycin and ciprofloxacin. In contrast, Product C does not demonstrate the presence of viable *L. crispatus* since correspondence is found only when comparing the total viable lactobacilli 8.0 ± 0.1 Log(CFU/gr) with those evaluated after adding vancomycin to MRS 7.5 ± 0.4 Log(CFU/gr). The two French products (D and E, where E corresponds to a MD) show good correspondence between the value obtained with MRS, respectively 8.1 ± 0.3 and 8.0 ± 0.2 Log(CFU/gr) and after adding clindamycin and ciprofloxacin respectively 8.1 ± 0.3 and 8.0 ± 0.3 Log(CFU/gr), demonstrating the likely presence of viable *L. crispatus*. Product F declares that it contains both an *L. crispatus* strain, which grows on MRS plus clindamycin and ciprofloxacin, and an *L. rhamnosus* strain, which grows on MRS plus vancomycin. Indeed, we have observed the growth of viable cells in both of the supplemented media, respectively 8.0 ± 0.3 and 9.7 ± 0.3 Log(CFU/gr). This demonstrates the likely presence in F of viable *L. crispatus*. Product G seems to contain a low count of lactobacilli growing on MRS 2.0 ± 0.1 Log(CFU/gr) and negative results were obtained in the two antibiotic-supplemented media. Product H, declaring that it contains *L. crispatus* and *L. rhamnosus*, demonstrates growth of viable lactobacilli on MRS 6.7 ± 0.2 Log(CFU/gr) and the same growth on a medium to which vancomycin has been added 6.7 ± 0.5 Log(CFU/gr) (likely to be *L. rhamnosus*) but the absence of growth on a medium selecting *L. crispatus.* Product I (a MD) shows the prevalence of *L. rhamnosus* and *L. reuteri* on MRS medium 11 ± 0.1 Log(CFU/gr) despite the presence of some *L. crispatus* colonies. However, the same colonies were unable to grow in the medium supplemented with clindamycin and ciprofloxacin suggesting the potential inability of this *L. crispatus* strain to grow on the selective medium. The medium supplemented with vancomycin confirmed the high presence of *L. rhamnosus* and *L. reuteri* strains with the recovery of viable counts of10 ± 0.4 Log(CFU/gr). A final comparison of the real viable counts obtained for the *Lactobacillus* genus and for the presumptive *L. crispatus* species with the labelled daily dose is reported in Table 3. The real daily dose was empirically calculated by dividing the viable count values for the suggested assumption dose. As shown, only five products (A, B, D, E, F) out of nine contain viable and quantifiable *L. crispatus* on the selective medium, while products C, G, H and I do not appear to do so. Considering instead the real daily dose potentially administrable by the oral route, Product A reaches the highest value (about 2.4 × 10^10^ CFU/gr *L. crispatus*), while Product B, the only product along with Product A attaining the value of 1 × 10^9^ CFU/gr of living cells, reaches a value that is one tenth the amount of *L. crispatus* with respect to Product A. Products D, E and F reach very low values, between 3.5 and 6.3 × 10^7^ CFU/gr viable *L. crispatus* per daily administrable dose.

### 3.2. 16 S rRNA Gene Amplification and Molecular Typing of Viable Colonies

**Figure 1:**
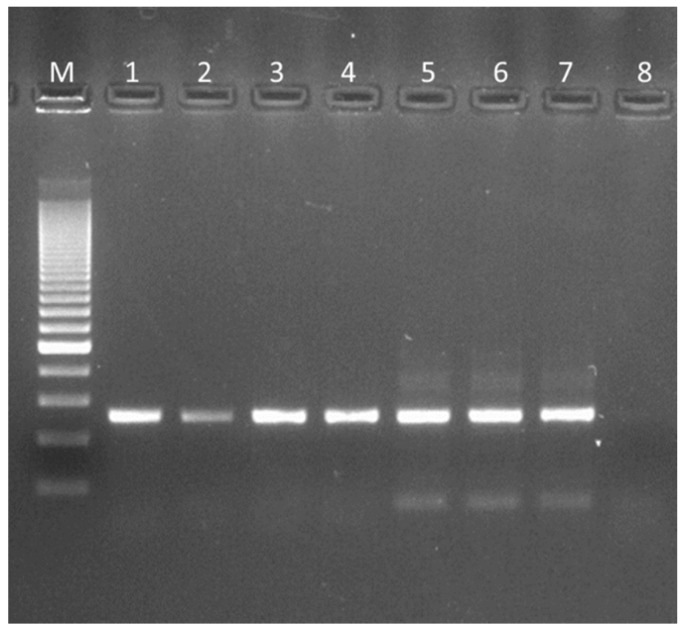
Specific PCR assay for *L. crispatus* 16S rRNA; positive results obtained from vital colonies grown on plates. Within the figure: Marker 200 bp (M); LMG 9479-*L. crispatus* type strain (1); Product A (2); Product B (3); Product D (4); Product E, MD (5); Product F (6); Product I, MD (7); negative template control (8).

**Figure 2:**
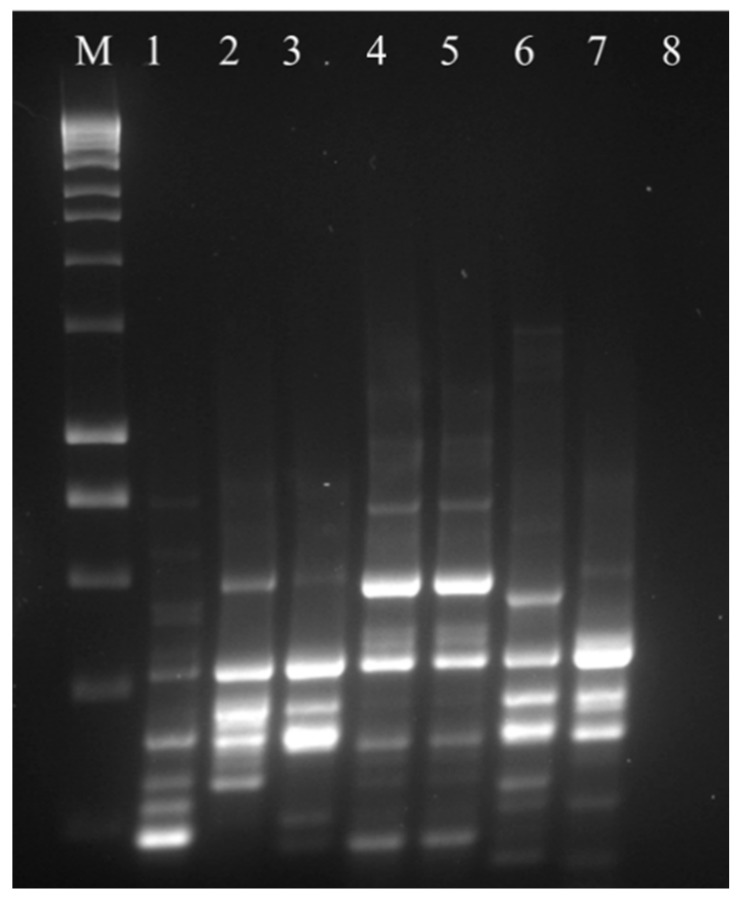
Molecular typing by RAPD-PCR of *L. crispatus* strains obtained by growth of vital colonies on plates. Within the figure: Marker 1 kb (M); LMG 9479-*L. crispatus* type strain (1); Product A (2); Product B (3); Product D (4); Product E, MD (5); Product F (6); Product I, MD (7); negative template control (8).

The products of amplification obtained with specific primers for *L. crispatus* are shown in Figure 1. The selection of colonies displaying *L. crispatus*-like morphology was performed both on MRS and MRS supplemented with 0.1 and 10 µg/mL clindamycin and ciprofloxacin. After random selection, and DNA extraction, only six out of the nine analyzed products displayed viable colonies belonging to this species. The typing approach with the RAPD2 primer is shown in Figure 2. The patterns obtained for the six isolated *L. crispatus* and for the positive control LMG 9479 are different and unique, underlying the individual molecular characteristics of these strains. 

### 3.3. 16 S rRNA Gene Amplification of the Finished Products

**Figure 3:**
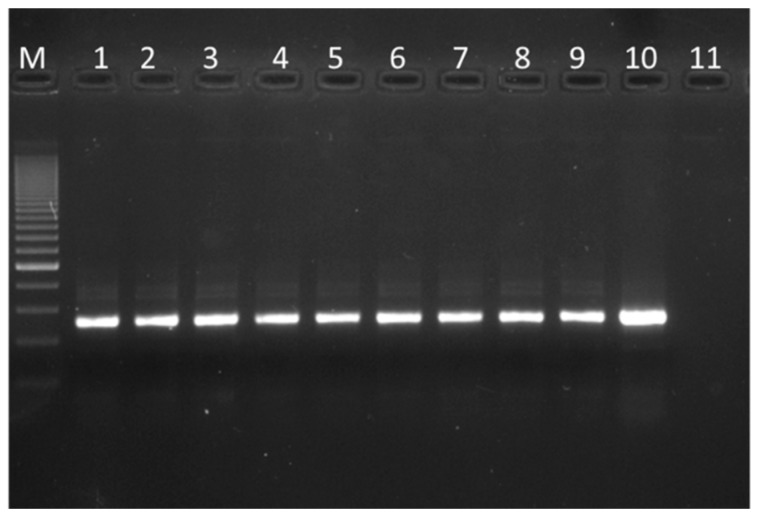
Specific PCR assay for *L. crispatus* 16S rRNA; total DNA extraction from powders. Within the figure: Marker 200 bp (M); LMG 9479-*L. crispatus* type strain (1); Product A (2); Product B (3); Product C (4); Product D (5); Product E, MD (6); Product F (7); Product G (8); Product H (9); Product I, MD (10); negative template control (11).

Since only six products contained viable colonies of *L. crispatus,* the total DNA was extracted from the finished dietary supplements and amplification of the 16S rRNA gene was performed with the same specific primers previously used for viable colonies. The PCR results are shown in Figure 3. All probiotic powders were positive with respect to amplification for *L. crispatus*, but it is likely that cells in products C, G, H and I had lost their viability or, with special refence to product I, are unable to grow in the selective medium supplemented with antibiotics.

## 4. Discussion

Medical practice indicates both local and oral administration as a possible way for a probiotic to reach the vaginal environment [23,24,25]. In any case, from 2020, the use of live probiotic strains for insertion into the vagina as MDs will no longer be allowed in Europe, unless they are registered as drugs [26]. The obvious difficulties in obtaining such a registration status for probiotics will determine in all likelihood the sunset of the vaginal use of probiotics, and most of the products available for gynecological use in the near future will be nutritional supplements suitable for oral use only. Therefore, in our study we have decided to analyze the quality of probiotics containing at least one strain of *L. crispatus* in formulations that have been notified to the Health Authority as nutritional supplements. We have found, between pharmacies and online, seven different nutritional supplements containing at least one strain of lactobacillus declared to be *L. crispatus*. We also found two vaginal suppositories, registered as an MD, claiming to contain one strain of *L. crispatus*. Even if these products will be most likely removed from the European market by 2020, for study completeness we decided to analyze it as well here.

The results that we have obtained describe a not entirely positive situation. First of all, commercial products in which *L. crispatus* is detectable, dead or alive (results obtained by qualitative PCR performed using the product powders) are all of the products (A, B, C, D, E, F, G, H, I) according to Figure 3. Second, commercial products in which *L. crispatus* is viable (PCR amplification from colonies retrieved from agar plates) corresponds, according to Table 2 and Table 3 and Figure 1, to products A, B, D, E, F and I. It has to be noticed that the viable colony of the *L. crispatus* strain of the product I was isolated starting from the MRS agar plate. Third, considering only the nutritional supplements (the MDs will soon be taken off the market), the products for which the total viable count truly corresponds to the label (quantitative plate count, see Table 2 and Table 3) are A, B and F. It should also be noted that, at the time of analysis, all nine products studied marked as being 18 months before the declared expiry date. Fourth, again considering only the food supplements, the products in which *L. crispatus* is found to be viable with an acceptable plate count are A and B only (Table 2 and Table 3) with Product A dosing 10 times more than Product B. In our opinion, our study confirms that a low-quality product profile frequently threatens the probiotic sector [4,5,6,7,8,9]. In terms of supplements containing a very high count of clearly viable *L. crispatus*, only Product A, containing the strain M247, gives satisfactory results. Notably, M247 (LMG P-23257) is the only strain that has been thoroughly described in the scientific literature. Studies reported that *L crispatus* M247 could influence the tissue Peroxisome Proliferator-Activated Receptor-gamma (PPAR-gamma) levels and modulate epithelial cell receptivity to inflammatory signals [27]. The strain was also investigated by means of proteomic approach to characterize its aggregation abilities strictly linked to the its adhesive features [28]. In vivo murine models it was also evaluated for its ability to colonize the colonic mucosa upmodulating Toll-like receptors 2 and 4 [29] and for its protective effect exerted during experimentally induced colitis [30]. The strain was also genetically described for its aggregation features [31] and it was also investigated for its intestinal mucus-binding properties and in terms of ability to restore the normal vaginal microbiota [32,33,34].

Differently, all the other strains used to formulate the other probiotic supplements studied by us failed to demonstrate to be scientifically described, at least within the PubMed (Table 4) or Google Scholar (data not shown) systems. Even more worrying, for Product I (one of the two MD analyzed), lactobacilli species are listed without declaring the strains used. As shown in Table 4, besides the *L. crispatus* strain M247 documented by seven papers, we found 10 papers on PubMed documenting the strain *L. crispatus* CTV-05. According to our information, this strain is traded in the USA and not in Europe as Lactin V (Osel Inc., Mountain View, CA, USA). Unfortunately, we did not obtain a sample of this finished product to analyze its quality before our study ended. We are therefore unable to comment on its quality. In any case, it has been indicated in Table 4 for the sake of completeness.

Nevertheless, our report sufficiently clarifies the issue of the “*L. crispatus* market”, although we have to acknowledge some apparent discordance and the limitations of our study. It is a fact that not all *L. crispatus* strains can grow on MRS supplemented with 0.1 and 10 µg/mL clindamycin and ciprofloxacin. This is the possible explanation for the negative/positive results regarding the presence of *L. crispatus* in Product I, one of the two MDs. Results were negative when observed in MRS supplemented with antibiotics and positive when genetic analysis was performed on MRS plates containing colonies displaying *L. crispatus*-like morphology. Furthermore, *L. crispatus* strains IMC607S (declared to be in Products C and G) and P17631 (declared to be in Product H) are definitely unable to grow on MRS medium supplemented with 0.1 and 10 µg/mL clindamycin and ciprofloxacin. Reference medium for the cultivation of *Lactobacillus* spp. supplemented with clindamycin and ciprofloxacin is highly specific for *L. acidophilus* and *L. crispatus* is only phylogenetically strict to that, but it is not exactly the same species. Lastly, we have to admit the lack of a molecular analysis that was able to quantify the presence of *L. crispatus* starting from the traded finished products. We attempted quantification with qPCR, but the assessment was not successful, probably due to the presence of maltodextrins and/or other inhibitors of DNA extraction and Taq enzyme amplification. In conclusion, our investigation highlights the low-quality profile of the probiotic dietary supplements containing *L. crispatus* strains with just two (Products A and B) out of eight products which could be considered acceptable in terms of correspondence of formula, correspondence of dosage with labelling and appropriateness in terms of administrable daily dose. Finally, just one product (A) out of eight has been formulated using a scientifically evaluated strain, this being the only strain (*L. crispatus* M247) documented, in human studies too, at least according to output from PubMed or Google Scholar and excluding data coming from patents, whose scientific evaluation certainly seems too complex and influenced by commercial aspects to be, in our opinion, contemplated in this context. 

## 5. Conclusions

The panorama of probiotic products labelled to contain at least one strain of *Lactobacillus crispatus* appears, according to our studies, to be rather poor in terms of microbiological quality. Only in one product the strain (M247) appears to be scientifically well-described and properly formulated in regards to daily dosage and viability.

## Figures and Tables

**Table 1:** Microbiological compositions and viable count values declared on labels.

Product	Pharmaceutical Form	*L. crispatus* Strain	Other Bacterial Species	Market	Total Dose Declared (CFU/gr)	*L. crispatus* Dose Declared (CFU/gr)
(A)	Stick	M247	/	Italy/NS	not less than 2 × 10^10^	not less than 2 × 10^10^
(B)	Sachet	LBV10	*L. gasseri* LBV162	Italy/NS	not less than 2 × 10^9^	not less than 1 × 10^9^
(C)		IMC6075	*L. rhamnosus* IMC501,	Italy/NS	4 × 10^11^	1 × 10^11^
	Capsule		*L. jensenii* IMC813S,			
			*L. reuteri* LR92			
(D)	Capsule	IP174178	/	France/NS	2.8 × 10^8^	2.8 × 10^8^
(E)		IP174178	/	France/MD	4 × 10^8^	4 × 10^8^
(F)		KS127	*L.rhamosus* LB21,	Italy/NS	2 × 10^10^	2 × 10^9^
	Capsule		*L. plantarum* LB931,			
			*B. lactis* Bi1			
(G)		IMC607S	*L. gasseri* LG050	Italy/NS	5.4 × 10^9^	2.7 × 10^9^
(H)	Sachet	P17631	*L. rhamnosus* ATCC53103	Italy/NS	ND	ND
(I)		Not indicated	*L. rhamnosus,*	Italy/MD	4 × 10^11^	1 × 10^11^
	Capsule		*L. jensenii,*			
			*L. reuteri*			

NS: nutritional supplement; MD: medical device.

**Table 2:** Logarithmic transformed mean values (CFU/g) of viable counts for three independent replicates and SD obtained for products on different media.

		Log 10(CFU/gr)		
Product	*L. crispatus* Strain	MRS	MRS+Clinda/Ciproflox	MRS+Vancomycin
(A)	M247	10.4 ± 0.2	10.4 ± 0.4	ND
(B)	LBV10	9.4 ± 0.1	8.9 ± 0.1	ND
(C)	IMC6075	8.0 ± 0.1	NG	7.5 ± 0.4
(D)	IP174178	8.1 ± 0.3	8.1 ± 0.3	ND
(E)	IP174178	8.0 ± 0.2	8.0 ± 0.3	ND
(F)	KS127	9.9 ± 0.1	8.0 ± 0.3	9.7 ± 0.3
(G)	IMC607S	2.0 ± 0.1	NG	NG
(H)	P17631	6.7 ± 0.2	NG	6.7 ± 0.5
(I)	NI	11 ± 0.1	NG	10 ± 0.4

NG = not growing; ND = not determined for products composed only of *L. crispatus* and/or *L. gasseri.* strains; NI = not indicated.

**Table 3:** Comparison of experimental viable counts (scaled to the weight per day) with the labelled daily dose for the total and *L. crispatus* viable counts expressed as 10^9^ (CFU/day).

Product	*L. Crispatus* Strain	Weight (g)	Daily Dose	Total probiotics-Measured Viability Per Daily Dose	Total probiotics-Declared Daily Dose	Potential *L. crispatus* Per Daily Dose	Declared *L. crispatus* Per Daily Dose
(A)	M247	1 g/stick	1 stick	10.4	10	10.4^1^	10
(B)	LBV10	2.8 g/sachet	1 sachet	9.7	9.3	9.2^2^	9
(C)	IMC6075	0.35 g/capsule	1 capsule	7.5	9.6	NG	9
(D)	IP174178	0.45g/capsule	1 capsule	7.8	8	7.8^3^	8
(E)	IP174178	0.35 g/capsule	1–2 capsule	7.5	9	7.5^4^	9
(F)	KS127	0.5 g/capsule	1 capsule	9.6	9.8	7.7^5^	9
(G)	IMC607S	0.375 g/capsule	1 capsule	1.5	9.3	NG	9
(H)	P17631	1.5 g/sachet	1 sachet	6.3	NL	NG	NL
(I)	NI	0.35 g/capsule	1 capsule	10.6	10.4	NG	9.7

NL = not labelled; NG = not growing; NI = not indicated; ^1^: corresponding to 24 × 10^9^ CFU/day; ^2^: corresponding to 2.1 × 10^9^ CFU/day; ^3^: corresponding to 63 × 10^6^ CFU/day; ^4^: corresponding to 35 × 10^6^ CFU/day; ^5^: corresponding to 50 × 10^6^ CFU/day (all values are calculated from the log scale value).

**Table 4:** Number of documents found on PubMed for the strains indicated in the nine products analyzed and those found on strain CTV-05 (product X, not analyzed).

Product	*L. crispatus* Strain	N
A	M247	7
B	LBV10	0
C/G	IMC607S	0
D/E	IP174178	0
F	KS127	0
H	P17631	0
I	Type of strain not indicated	N. F.*
X	CTV-05	10 ^

* Information impossible to obtain as the strain is not indicated in the finished product. ^ According to our information, the strain is used in the USA and traded in a product named Lactin V by Osel Inc., California, CA, USA.
